# Interference of phototherapy with blue LED light on the behaviour of mice infected with *Toxoplasma gondii*

**DOI:** 10.1371/journal.pone.0353740

**Published:** 2026-07-14

**Authors:** Marina Monteiro de Castro Burle, Ben-Hur Araújo Batista da Silva, Débora Nonato Miranda de Toledo, Lauro de Assis Duarte Junior, Rodrigo Fernando Bianchi, Érica S. Martins-Duarte, André Talvani, Cristiano Schetini de Azevedo

**Affiliations:** 1 Department of Biodiversity, Evolution, and Environment, Universidade Federal de Ouro Preto, Ouro Preto, Minas Gerais, Brazil; 2 Department of Biological Sciences, Universidade Federal de Ouro Preto, Ouro Preto, Minas Gerais, Brazil; 3 Faculty of Medicine, Universidade Federal de Minas Gerais, Belo Horizonte, Minas Gerais, Brazil; 4 Department of Physics, Universidade Federal de Ouro Preto, Ouro Preto, Minas Gerais, Brazil; 5 Department of Parasitology, Universidade Federal de Minas Gerais, Belo Horizonte, Minas Gerais, Brazil; National Institute of Child Health and Human Development (NICHD), NIH, UNITED STATES OF AMERICA

## Abstract

*Toxoplasma gondii* is an intracellular protozoan capable of promoting physiological and behavioural changes in hosts. When female mammals acquire *T. gondii* for the first time during pregnancy, Congenital Toxoplasmosis (CT) can occur, posing a significant risk to the foetus. Due to challenges in diagnosing and treating CT, a new blue LED light therapy (BLLT) was proposed to eliminate parasites during placenta and tissue invasion in mice; however, its effects on the behaviour of the animals are unknown. Thus, behavioural analysis was carried out in pregnant infected Swiss mice under BLLT (applied continuously for 12 hours, from 7 am to 7 pm, maintaining an intensity of 460 nm and 7 μW/cm²). Infected mice, regardless of light exposure, exhibited increased inactivity and reduced maintenance behaviours. BLLT influenced certain behaviours, such as an increase in abnormal behaviours in infected mice and higher food and water intake in non-infected mice, suggesting a potential stress effect. Grooming decreased under BLLT in infected mice, while affiliative interactions were reduced in non-infected mice under conventional light. Activity levels were largely unaffected in infected mice, but blue light exposure increased activity in non-infected mice. While our study highlights the potential of BLLT to reduce parasite load, further research is needed to investigate the long-term behavioural and physiological effects, as well as to clarify the mechanisms involved. In conclusion, BLLT may mitigate parasite load and influence behavioural outcomes, while prolonged exposure can act as a stressor, moderately affecting welfare. Future research should explore optimized light regimens, integrate physiological and behavioural measures, and evaluate long-term effects to balance therapeutic efficacy and animal welfare.

## Introduction

Parasitism is an ecological interaction where parasites cause metabolic and physiological changes in hosts [1,2]. During this interaction, the parasite can also promote behavioural changes in the host, which can be general and/or specific [3]. General changes are typically associated with the immune system and its response to an infection, with behaviours such as prostration, reduced search for and ingestion of food and water, and reduced maintenance behaviours being observed [4–7]. As for specific ones, they are observed through the manifestation of changes in the frequency and/or intensity of natural behaviours or through the presentation of abnormal and/or non-specific behaviours, capable of increasing the chances of propagation and closing the biological cycle of the parasite itself [8–10]. These more specific behavioural changes serve as the basis for a hypothesis that is discussed among many scholars, the so-called “Behavioural Manipulation Hypothesis,” in which it is believed that the parasite can manipulate the host’s behaviour to improve its reproductive fitness [8,11–14].

One of the parasites that stands out in this hypothesis is *Toxoplasma gondii*, the causative agent of toxoplasmosis [11,15,16]. During *T. gondii* infection, behavioural changes have increased the chances of its transmission via copulation through changes in sexual behaviour between intermediate hosts [17,18] and trophic transmission through changes in self-defence and risk behaviours that make the intermediate host more prone to predation by the definitive host [4,9,19]. The behavioural changes during toxoplasmosis have already been observed in various species of intermediate hosts, such as grey wolves (*Canis lupus*) [19], chimpanzees (*Pan troglodytes troglodytes*) [20] and even humans [21].

*T. gondii* is an obligate intracellular protozoan capable of infecting various warm-blooded mammals and birds [22–26]. Despite having a wide variety of intermediate hosts, where only asexual reproduction occurs, only felids (domestic and wild cats) are its definitive hosts, where the parasite reproduces sexually [11,22,25]. *T. gondii* has a global distribution, and since its first description by Nicolle and Manceaux [27] and Splendore [28], toxoplasmosis has been recognised as a zoonosis of great importance [24,29,30]. When an acute infection occurs during the host’s gestation period, vertical transmission is possible, allowing parasites to cross the placenta and infect the embryo/fetus [25,31,32], resulting in Congenital Toxoplasmosis (CT) [11,22,26,33].

CT is a disease with a high impact on public health, affecting women all over the world, mainly in low-income countries [34–36]. An aggravating factor is that the mother is often asymptomatic or has non-specific symptoms, which can be mistaken for various less serious illnesses [23–25,37,38]. This situation is particularly concerning, as it often hinders timely diagnosis and the implementation of effective early treatment [31]. The current allopathic therapeutic approach for CT depends on the condition and stage of the infection, and it is necessary to know the age of the pregnancy and when transmission to the fetus occurred [39]. Current standard treatment protocols primarily involve administering spiramycin during early pregnancy to reduce the risk of vertical transmission, whereas combinations of pyrimethamine, sulfadiazine, and folinic acid are generally recommended after confirmed fetal infection or during later gestational stages [39,42,43]. Although these regimens can reduce parasite transmission and disease severity, they do not eliminate *T. gondii* tissue cysts and often require prolonged administration [23,40]. To make a diagnosis of congenital transmission, it is necessary to carry out specific tests, such as PCR of the amniotic fluid and an ultrasound [41], however, these tests do not provide enough information to guarantee the effectiveness of the treatment [31,32,39]. In addition to these limitations, the therapeutic protocols used have several disadvantageous factors [42], including potential teratogenicity, nephrotoxicity, hepatotoxicity, and myelotoxicity [32,40,43,44]. Furthermore, currently available drugs cannot eliminate *T. gondii* from host tissues, allowing latent forms to persist and potentially reactivate under immunosuppressive conditions [41]. Prolonged treatments and adverse effects may also compromise patient adherence, especially in congenital toxoplasmosis cases requiring long-term therapeutic management [40,44]. Alternative therapies are demanded to support the clinical care of pregnant women [23,40].

One alternative therapy is the blue LED light therapy (BLLT). In 1958, the blue-violet light spectrum gained attention for its effectiveness in treating juvenile jaundice [45]. Since then, several studies have shown that BLLT is an effective tool for treating not only metabolic diseases but also bacterial diseases [46–48], fungal diseases [47], and potentially viral diseases [46]. More recently, antimicrobial light-based therapies have also been investigated as adjunct approaches for protozoan infections due to their potential immunomodulatory and antiparasitic effects and reduced systemic toxicity [42,47,48,51]. A recent study demonstrated the effectiveness of BLLT in reducing parasite load during *in vivo* infection with *Trypanosoma cruzi* [47]. Similarly, experimental evidence suggests that blue light exposure may interfere with parasite replication and inflammatory responses, supporting its investigation as a potential complementary therapy for toxoplasmosis [42,51].

In addition to assessing the effectiveness of a treatment, it is essential that an *in vivo* experiment also considers the level of well-being and potential factors that negatively interfere with the physiology and behaviour of the experimental animal. Chronic stress [48], for example, can trigger profound physiological changes and an imbalance in the immune response [49,50]. Many experimental results can be obscured by secondary factors that alter the environment and the animal’s behaviour during confinement or experimentation [51–53]. The light to which the animal is exposed also plays an essential role in metabolic regulation and homeostatic physiology [54], and it can modify the capacity of the organism to respond to stress [54]. In this context, assessing the well-being of animals undergoing phototherapy is paramount. Considering that a stressful stimulus triggers three types of responses in the individual’s body: neuroendocrine (activation of the hypothalamic-pituitary-adrenal axis), autonomic (Sympathetic Nervous System), and behavioural, it is possible to assess the conditions and consequences of exposure to stress by evaluating the parameters involved in these responses [48,49,55]. Therefore, behavioural observation is a valid approach that can provide substantial information about animal welfare in a non-invasive way [56,57].

In rodents, stress can stimulate aggressive behaviour, generating more conflicts between confined individuals and, depending on the intensity/frequency, the risk of injury, severe damage, and even death [58]. Rodents with toxoplasmosis exhibit greater exploration of the environment, no avoidance of adverse stimuli (such as light, for example), less neophobia, and a specific attraction to the smell of feline urine [11–13,59,60]. In the case of mice, it can be more active, with less preference for exposed areas [61], can also run in circles, present head tilt [10], and reduce specific behaviours, such as “rearing” (characteristic environmental exploration/search behaviour with head up to investigate the presence of more distant stimuli [62]), digging behaviours [63,64], and present lower learning capacity and reduced short-term memory [65]. Thus, behavioural observation of these animals is a useful tool for assessing both stress conditions and the progression of *T. gondii* infection, as specific behavioural changes associated with these conditions are observed.

Although there are already studies on the behaviour of animals under treatment with BLLT [66–68], there are no reports evaluating the behaviour of animals submitted to treatment while infected with *T. gondii*. Therefore, the purpose of this study is to assess possible behavioural changes in mice infected with *T. gondii* under treatment with BLLT and determine whether the treatment in question is recommended based on the behaviours expressed by the animals.

We hypothesise that the treatment with BLLT will reduce the behavioural changes caused by *T. gondii* infection, making it possible to observe mild or almost zero changes in mice behaviour when comparing the treated infected group to the control group. At the same time, untreated infected animals will exhibit specific behavioural changes characteristic of the infection, including changes in activity and the display of abnormal behaviour, indicating the possible effectiveness of BLLT as an alternative therapeutic approach for toxoplasmosis.

## Materials and methods

### Parasite

The ME49 strain of *T. gondii*, classified as type II and avirulent [69], was obtained from the Egler Chiari Protozoan Chemotherapy Laboratory at the Federal University of Minas Gerais (UFMG). Cysts of the ME49 strain were obtained from the brain tissues of chronically infected mice, as previously described by Martins-Duarte et al. [70]. Briefly, brain tissues from chronically infected donor mice were mechanically homogenised in sterile saline solution, and cysts were counted under light microscopy. For the oral infection, each experimental female received a suspension containing exactly 10 cysts by gavage after confirmation of mating. This low-dose oral inoculation protocol was selected to reproduce congenital infection while minimising excessive mortality and distress during pregnancy.

### Animals, housing, and maintenance

The experiment was carried out at the Animal Science Centre of the Federal University of Ouro Preto (UFOP) and used 18 female Swiss Webster mice (8–9 weeks old) (n = 18) kept in an environment with a light cycle (12h/12h light/dark cycle) and controlled temperature (22.0 ± 2°C), with water and food *ad libitum*. The boxes were inspected every two days, the wood shavings were replaced, and the ration and water were replenished as needed. To better visualise the filming, the ration was spread out inside the box, and the bottle was positioned in a hole on the side. The animals were also weighed to assess pregnancy condition and overall health. The study was approved by the UFOP Research Ethics Committee (CEUA) (No. 3243161121), and it is under Brazilian regulations by CONCEA [71] and in compliance with ARRIVE guidelines [72,73].

### Experimental design and phototherapy

**Fig 1:**
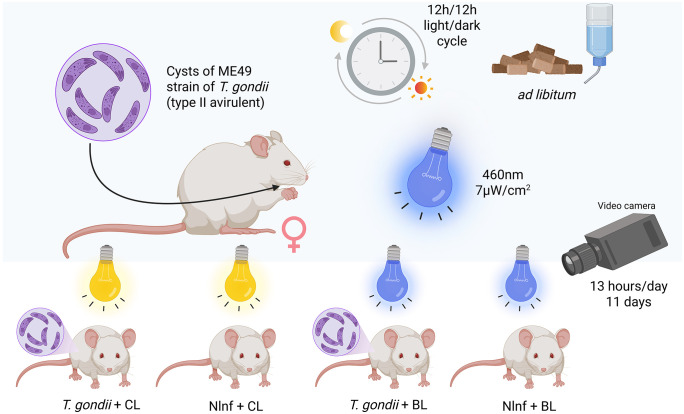
Schematic representation of the experimental design. Following mating (2:1 female-to-male ratio for 7 days) and confirmation of copulation via vaginal plug detection, females were either infected with 10 cysts of the *Toxoplasma gondii* ME-49 strain or left uninfected. Animals were then assigned to four groups: infected exposed to conventional light (*T. gondii* + CL; n = 5), infected exposed to blue light (*T. gondii* + BL; n = 5), uninfected exposed to conventional light (NInf + CL; n = 4), and uninfected exposed to blue light (NInf + BL; n = 4). Blue light phototherapy was administered for 12 hours daily during the light phase (07:00–19:00) at 460 nm and 7 μW/cm², from the first day of infection until euthanasia on day 11. This figure was created by the author MCB using Canva’s Pro Content library, which is licensed for use without restriction for online publications according to Canva’s Content License Agreement (Terms for Pro Content; Sections 2 and 3, Free Content License).

The females were housed with males at a 2:1 ratio for seven consecutive days to allow natural copulation. Pregnancy was confirmed by the presence of a vaginal plug at the end of this period. Although the exact gestational day at which the infection occurred cannot be precisely determined, it is estimated to have occurred within the first week after copulation. Following confirmation of mating, the females were orally infected with 10 cysts of the ME-49 strain of *T. gondii*, as described by Martins-Duarte et al. [70]. This approach minimised handling stress and interference with mating behaviour while maintaining a reasonably consistent infection window during early gestation. For the experiment, animals were grouped into infected mice exposed to conventional light (n = 5), infected mice exposed to BLLT (n = 5), uninfected mice exposed to conventional light (n = 4), and uninfected mice exposed to BLLT (n = 4) (Fig 1). To confirm infection and assess potential differences in parasite load between infected individuals exposed and unexposed to BLLT, real-time quantitative PCR (qPCR) analyses were performed [74]. Infection was confirmed in all infected groups, and reduced cerebral parasite burden was observed in animals exposed to BLLT. The complete methodological description and detailed qPCR results have been previously published in Toledo et al. (2026) [75], which analysed parasitological and oxidative stress parameters from the same experimental trial. To avoid unnecessary duplication of previously published material, only a summary of these findings is presented in the S1 File Supporting Information.

Phototherapy was applied using a device specifically developed for this study by the team from the Laboratory of Materials Physics at the Federal University of Ouro Preto (S2 File Supporting Information). The BLLT was applied continuously for 12 hours during the light phase of the standard 12 h/12 h light–dark cycle (from 7:00 am to 7:00 pm), maintaining an incident blue light of 460 nm and 7 μW/cm² (Fig 1). The irradiance at cage level was measured using a calibrated radiometer (Fanem Multitester Thor® 3620). Calibration followed manufacturer specifications, ensuring reliable and reproducible measurements before and during the experimental period. The distance between the LED source and the cages was standardised at 32 cm. This setup was ensured by a custom-made experimental apparatus that kept the LEDs vertically aligned and stable relative to the cages. This distance was maintained throughout the experiment, guaranteeing uniform irradiance for all exposed animals. In addition, an opaque physical barrier separated the illuminated compartment from the non-illuminated control area, ensuring that animals in the conventional-light groups were not exposed to residual blue light (see S2 File Supporting Information).

Temperature was carefully monitored to prevent confounding effects from heat generated by the LEDs. Surface and cage temperatures were periodically measured using a portable infrared laser thermometer (−50 °C to 1000 °C, adjustable emissivity), and fans were positioned near the lamps to dissipate excess heat, thereby maintaining equivalent thermal conditions between the experimental and control groups. Because the irradiance was extremely low (in the μW/cm² range), the exposure did not generate measurable heat. Control groups were housed in a separate, identical environment under the same light–dark cycle and ventilation conditions, with LED lamps turned off to ensure no residual light or heat exposure. The light source consisted of Ourolux SuperLed Colors 6W BiVolt (Blue) lamps, which emit predominantly at ~460 nm. According to the manufacturer’s specifications, these LEDs exhibit high spectral stability, ensuring that the exposure remained confined to the intended blue wavelength range throughout the experiment.

The device was manually switched on and off at the same time each day to ensure that exposure occurred exclusively during the daytime phase, with no irradiation provided during the dark period. This timing was chosen to avoid extending the photoperiod or introducing light at night, both of which are known to cause greater circadian disruption in nocturnal rodents. Because mice are primarily active during the dark phase [80], restricting treatment to the light phase minimised potential interference with normal circadian entrainment and ensured that all groups experienced identical light–dark conditions, except for the daytime blue-light treatment. Although blue light may still modulate physiological rhythms, this design reduces the likelihood of major circadian confounding effects.

Treatment began on the first day of infection and ended when animals were euthanised, totalling 11 days of experimentation. The euthanasia of 14 females occurred using intraperitoneal sedation with ketamine (300 mg/kg) and xylazine (30 mg/kg). Euthanasia followed the Brazilian Guidelines for the Care and Use of Animals for Teaching and Scientific Research [71], where the animals were induced into deep anaesthesia using a fast-acting injectable agent, followed by exsanguination via cardiac puncture.

The experimental design used predefined terminal endpoints for all animals at specific gestational time points (e.g., the 12th day). This approach was scientifically necessary to comprehensively evaluate the effects of blue LED phototherapy on various parameters in a congenital toxoplasmosis model at critical stages of pregnancy. Furthermore, the ME49 strain of *T. gondii*, classified as type II and avirulent, which promotes a brand infection [76], was administered at a low dose of 10 cysts per mouse to minimise pain and distress for the animals. Animal health was monitored every three days by assessing body mass. Four animals died overnight during the last two days of data collection (two in the *T. gondii* + BL group and two in the *T. gondii* + CL group). As continuous monitoring was not feasible during these hours, the exact cause of death could not be determined. However, the timing is consistent with the acute phase (peak) of *T. gondii* infection. Pregnant mice are known to exhibit increased susceptibility to *T. gondii* infection due to gestational immune modulation, which may intensify acute disease manifestations, even with relatively low-virulence strains such as ME49 [76]. Therefore, although the inoculum was intentionally kept low (10 cysts/animal) to minimise excessive morbidity, some degree of mortality during the acute phase remained biologically plausible within this congenital infection model. Nonetheless, during regular daytime behavioural observations, no severe signs of distress or pain were noted that would have prompted early euthanasia. The animals only exhibited typical signs of infection, such as mild lethargy and ruffled fur. All behavioural data collected from these animals before death were retained and included in the statistical analyses. This procedure ensured transparency in data handling and avoided unnecessary exclusion of valid observations.

Animal welfare was a continuous priority throughout the study. Daily behavioural monitoring served as a key indicator of potential suffering or distress. While the study utilised pre-defined experimental endpoints for all animals, a strict humane endpoint protocol was also in place for severe, unexpected adverse events. Animals exhibiting clear signs of deterioration, such as significant weight loss (e.g., 15–20% of initial body weight), changes in general appearance (e.g., ruffled fur, hunched posture, or ocular/nasal secretions), severe abnormal behaviours (e.g., persistent lethargy, unresponsiveness, or uncoordinated movements), or an inability to eat or drink, would have been immediately and humanely euthanised to prevent undue suffering.

### Behavioural analysis

To evaluate the behaviour of the mice, we used an ethogram based on studies already published [77,78] and on a website developed by Stanford University on Mouse Ethograms [79] (Table 1).

**Table 1:** Ethogram used in the study of Swiss Webster mice (*Mus musculus*), based on a literature review [77–79].

Behaviour	Code	Description
**Grooming**	GRO	Usually in a sitting position, the mouse will lick its fur, groom with the forepaws, or scratch with any limb.
**Inactive**	INA	The absence of movement, i.e., the mouse is asleep or simply lying down.
**Affiliative Interactions**	AFI	Non-aggressive social interactions between cage mates, predominantly allogrooming and close social contact.
**Agonistic Interactions**	AGI	Non-affiliative social interactions between cage mates, including threatening behaviour, chasing, or fighting episodes.
**Abnormal Behaviour**	ABN	Stereotypic behaviours are repetitive and fixed in posture and behavioural sequencing, with no apparent function [e.g., circling (repetitively traces a loosely circular path, usually on the cage top) and bar-mouthing (is a bout of sham-biting on a fixed area of the cage bars)].
**Active**	ACT	General locomotor and exploratory activities, including walking, climbing, and environmental exploration.
**Drinking**	DR	Drinking water from the bottle.
**Feeding**	FE	Eating the food.
**Attend**	ATT	The mouse, from a short distance away, interrupts ongoing locomotion and fixes its head, eyes and ears toward a discrete stimulus, for a minimum of ~1–2 seconds, before either (i) approach or investigation of the stimulus or (ii) resumption of locomotion.
**Other Behaviours**	OTH	Rare or infrequent behaviours not encompassed by the predefined ethogram categories, including atypical interactions with conspecific corpses (e.g., investigation, contact, or cannibalism), prolonged object manipulation, and other isolated behaviours observed sporadically during the experiment.
**Not Visible**	NV	At the time of data collection, the behaviour was not identified.

To collect behavioural data, animals were filmed using CCTV surveillance cameras with night vision. Behavioural observations were conducted manually by trained observers, without the use of automated tracking software. Behavioural data were recorded from the footage on previously prepared field sheets using the scan sampling method with instantaneous recording at predefined 30-second intervals during the first 10 minutes of each hour [80,81]. For each scan, the presence or absence of predefined behaviours (e.g., grooming) was noted, and behavioural measures were subsequently quantified as total counts of occurrences across the observation period. The animals were recorded for 13 hours (06:30–19:30) until the 11th day of the experiment. Euthanasia was carried out the next day.

### Statistical analysis

The behavioural data generated were compiled in spreadsheets and analysed using descriptive statistics. The Anderson-Darling normality test was applied to the data to assess the distribution using the ‘nortest’ package [82]. Generalised linear mixed models (GLMMs) were built, with the recorded behaviours as response factors, the treatments (infected with *T. gondii* exposed to conventional light – “*T. gondii* + CL”; non-infected exposed to conventional light – “Ninf + CL”; infected with *T. gondii* exposed to the BLLT – “*T. gondii* + BL”; non-infected exposed to the BLLT – “NInf + BL”) as explanatory factors, and the day of the experiment as a random factor. GLMMs were built using the package ‘lme4’ [83]. Residual over-dispersion was evaluated using the package ‘performance’ [84]; all behaviours exhibited significant overdispersion (p < 0.05), confirming that negative binomial distribution was appropriate over Poisson. For low-frequency behaviours (Abnormal, Attend, Drinking, and Other), which showed high proportions of zeros (79–94%), we additionally compared standard negative binomial models with zero-inflated negative binomial (ZINB) models using the ‘glmmTMB’ package [85]. Model convergence and singularity were assessed using the ‘performance’ package [84]. A post-hoc Tukey test was carried out using the ‘emmeans’ package and adjusted for Sidak for pair-wise comparisons using the ‘multcomp’ and ‘multcompView’ packages. Exact p‑values, effect sizes (marginal and conditional R²), and 95% confidence intervals for rate ratios were calculated. Data from animals that died before the end of the study were included in all analyses up to the last day on which they contributed valid observations. All statistical analyses were conducted in R version 3.4.2 [86], with additional packages ‘DHARMa’ [87] for residual diagnostics and ‘glmmTMB’ [85] for zero-inflated model comparisons. The level of statistical significance was set at 95%.

## Results

A total of 55,531 behavioural records were collected. In all groups, inactive, active, and grooming behaviours were the most frequently displayed (S3 File Supporting Information). In the groups of non-infected animals, while in the group exposed to conventional light (NInf + CL), the least recorded behaviours were agonistic interactions, other behaviours, and drinking, respectively; in the group exposed to BLLT (NInf + BL), they were attending behaviour, other behaviours, and agonistic interactions (S3 File Supporting Information). As for the infected animals, in the group exposed to conventional light (*T. gondii*+CL), the least observed behaviours were, respectively, abnormal behaviours, attending behaviour, and agonistic interactions, while in the group exposed to BLLT (*T. gondii*+BL) they were attending behaviour, agonistic interactions, and abnormal behaviours (S3 File Supporting Information). “Not Visible” was not recorded during the experiment. To facilitate interpretation across multiple behavioural categories, an overview heatmap summarising relative behavioural frequencies across treatments is provided in S4 File Supporting Information.

**Fig 2:**
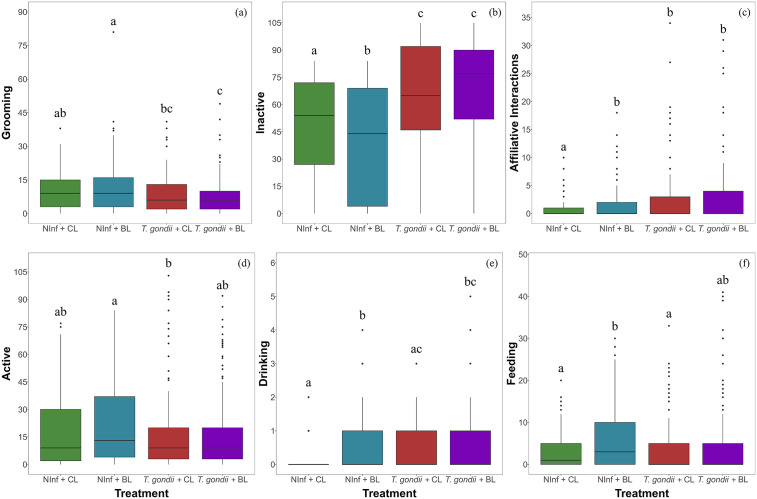
Behavioural records of “grooming”. (a), “inactive” (b), “affiliative interactions” (c), “active” (d), “drinking” (e), and “feeding” (f) displayed by the mice during the experiment, highlighting the treatments (NInf + CL: non-infected exposed to conventional light, n = 4; NInf + BL: non-infected exposed to BLLT, n = 4; T. gondii + CL: infected with T. gondii exposed to conventional light, n = 5; T. gondii + BL: infected with T. gondii exposed to BLLT, n = 5). Behavioural data were analysed using Generalised Linear Mixed Models (GLMMs), with treatment as a fixed factor and experimental day as a random factor. Significant treatment effects were observed for all behaviours shown (GLMM, p < 0.05). Boxplots: The dark line in the centre of the box represents the median, while the lower and upper edges of the box represent the first and third quartiles. The whiskers depict the range of the data, excluding outliers (shown as black dots). Letters indicate post hoc pairwise comparisons based on estimated marginal means (emmeans) with Sidak adjustment for multiple comparisons. Groups sharing at least one letter are not significantly different, whereas groups with different letters differ significantly (adjusted p < 0.05).

The GLMM results showed that Grooming decreased significantly in mice exposed to BLLT when infected (Fig 2a). Inactivity increased significantly in infected mice (both those exposed to conventional light and those exposed to BLLT) (Fig 2b). Affiliative interactions were less observed in the non-infected mice exposed to conventional light (named “control group” hereafter), with a significant difference compared to the other groups (Fig 2c).

Regarding activity level, none of the infected mice showed a significant difference from the control group. Still, a significant difference was observed between non-infected mice exposed to BLLT and infected mice exposed to conventional light (Fig 2d). The BLLT positively influenced the expression of drinking (Fig 2e) and feeding (Fig 2f) behaviours, with a significant increase observed in non-infected mice exposed to BLLT compared to the control group and in infected mice exposed to conventional light.

**Fig 3:**
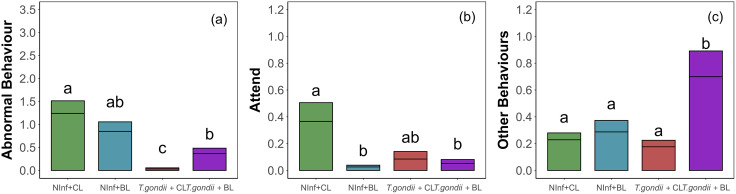
Behavioural records of “abnormal behaviour”. **(a), “attend” (b), and “other behaviours” (c) displayed by the mice during the experiment, highlighting the treatments (NInf + CL: non-infected exposed to conventional light, *n* = 4; NInf + BL: non-infected exposed to BLLT, *n* = 4; *T. gondii* + CL: infected with *T. gondii* exposed to conventional light, *n* = 5; *T. gondii* + BL: infected with *T. gondii* exposed to BLLT, *n* = 5).** Behavioural data were analysed using Generalised Linear Mixed Models (GLMMs), with treatment as a fixed factor and experimental day as a random factor. Significant treatment effects were observed for all behaviours shown (GLMM, p < 0.05). The bars represent the mean frequency of behaviours for each treatment group, while the horizontal dark line inside each bar represents the median value. Letters indicate post hoc pairwise comparisons based on estimated marginal means (emmeans) with Sidak adjustment for multiple comparisons. Groups sharing at least one letter are not significantly different, whereas groups with different letters differ significantly (adjusted p < 0.05).

Abnormal behaviours were significantly less observed in infected mice exposed to conventional light; however, in infected mice exposed to BLLT, this factor was a significant contributor to their display (Fig 3a). Attend behaviour was negatively influenced by phototherapy, with a significant difference between the BLLT-exposed groups (infected and non-infected) and the control group (Fig 3b). Finally, “other behaviours” were observed in all groups, with a significant difference between the infected mice exposed to conventional light and those exposed to BLLT (Fig 3c). This category mainly included atypical interactions with conspecific corpses (e.g., investigation and cannibalism), which occurred predominantly in the *T. gondii*+BL group. Detailed GLMM results, including exact p-values, effect sizes (marginal and conditional R²), and rate ratios with 95% confidence intervals, are provided in S5 File Supporting Information.

## Discussion

The study’s hypothesis was refuted because the infected mice treated with BLLT (*T. gondii*+BL) did not exhibit behaviour similar to the control group (NInf + CL), and the infected mice exposed to conventional light (*T. gondii*+CL) showed no specific behavioural changes characteristic of *T. gondii* infection. Specific behavioural changes caused by *T. gondii* infection are typically observed during the chronic phase of the disease [11], which may take weeks to develop, depending on the host’s immune response [59]. During the observation period of this study, the infected mice exhibited general behavioural changes that could be associated with sickness behaviour, a set of behavioural manifestations that occur during the acute phase of infections caused by various pathogens [5]. Sickness behaviour is mainly characterised by prostration (increased inactivity) and reduced maintenance behaviours (e.g., grooming) [6], which were observed in the infected mice, possibly reflecting the innate host response to infection [88]. The duration and intensity of the disease’s acute phase may be related to pregnancy status. Pregnancy is a factor that not only increases susceptibility to *T. gondii* infection but also worsens disease outcomes through immune responses during this period, thereby increasing the likelihood of sickness behaviour [89]. In congenital toxoplasmosis models, gestation itself represents a physiologically vulnerable condition because maternal immune modulation during pregnancy may increase susceptibility to systemic inflammatory responses while simultaneously affecting parasite control [76]. Depending on the timing of infection and host response, acute manifestations may include lethargy, reduced activity, ruffled fur, impaired maternal condition, and, in some cases, mortality during the peak of infection [75]. Thus, the mortality observed in the present study is biologically compatible with the known course of acute toxoplasmosis during pregnancy, particularly considering that deaths occurred in both infected groups and near the end of the observation period. Furthermore, it has already been observed that the acute phase is more pronounced in mice [10,90], which corroborates the difficulty of observing specific behavioural changes characteristic of *T. gondii* infection during the experiment.

The reduction in inactivity observed in BLLT-exposed mice may be related to the potential effects of light on circadian activity patterns [91]. *Mus musculus* is a crepuscular/nocturnal species, meaning its activity primarily occurs during the dark phase, leaving most of the light phase inactive. Within its time-activity budget, activity is observed for less than 50% of the time [92]. The wavelength of light to which the individual is exposed has a direct and indirect influence on their behaviour and physiology [93]. Blue light (470nm) can cause hormonal changes in mice, such as increased corticosterone levels, as well as behavioural changes, including increased arousal and consequent difficulty sleeping [94], and may modulate activity patterns. Although we did not directly measure circadian markers in the present study, these behavioural shifts are compatible with light-driven modulation of daily rhythms. Importantly, these findings are consistent with previous work in non-infected rodent models showing that blue-light exposure can modulate immune-related and endocrine pathways even in the absence of pathogens. Experimental studies have reported changes in stress hormone release, oxidative balance, and inflammatory mediators following short- or long-term exposure to blue wavelengths, indicating that light itself can act as a physiological regulator or stressor depending on the exposure regime [75]. Thus, the behavioural and parasitological outcomes observed here are consistent with the broader literature suggesting that photic stimulation may influence host immunity indirectly, in addition to potential pathogen-specific mechanisms.

The BLLT and *T. gondii* infection may interact to increase the animals’ overall physiological demands, pushing them further from homeostasis. In this context, the behavioural profile observed in *T. gondii*+BL mice could reflect attempts to restore or regulate internal balance. For instance, the increase in allogrooming, the affiliative behaviour most frequently observed, is consistent with its known role in reducing social tension and promoting relaxation in conspecifics [95]. Thus, heightened allogrooming may represent a compensatory strategy used by infected mice under BLLT to mitigate stress associated with their condition [94,96].

Conversely, the relative reduction in self-grooming observed in this group, although not statistically different from that in infected mice without BLLT, may indicate that the combined burden of infection and treatment has shifted behavioural priorities. During acute toxoplasmosis, sickness behaviours are expected and include decreases in energetically costly maintenance behaviours such as grooming. Therefore, the modest decline in self-grooming is likely driven primarily by infection-related malaise rather than by BLLT itself. Still, the interaction between these two factors may shape how animals allocate effort between maintenance and social behaviours.

An additional unexpected finding was the reduction in affiliative interactions in the non-infected mice kept under conventional light (NInf + CL). Unlike the groups exposed to blue light or infection, these animals did not present obvious physiological or environmental causes that would predict a decline in affiliative behaviour. One possibility is that the high proportion of inactivity recorded in this group, potentially linked to circadian suppression under conventional white light, may have passively reduced opportunities for social engagement. However, because this pattern was not consistently observed across groups and conventional light is generally considered a baseline housing condition, we cannot rule out the possibility that this represents an anomalous result influenced by uncontrolled microenvironmental factors (e.g., box hierarchy, subtle stressors, or stochastic variation). Thus, this finding warrants further investigation in future studies.

The reduction in abnormal behaviours may be related to the greater inactivity observed in infected individuals; however, exposure to BLLT increased abnormal behaviours in these individuals, possibly linked to light’s influence on the stress response to infection [54,67]. Given that the display of abnormal behaviours is correlated with low levels of well-being and increased stress [54,100–102], this finding is consistent with an interaction between infection and blue light exposure that affects stress-related behavioural expression in mice. It is also important to consider that pregnancy itself increases physiological and immunological demands in this model, potentially contributing to greater vulnerability to acute infection outcomes and behavioural instability during the experimental period.

Considering the active behaviour, the difference observed between the non-infected mice exposed to BLLT (NInf + BL) and the infected mice exposed to conventional light (*T. gondii*+CL) is probably related to the level of inactivity observed. In the case of the NInf + BL mice, the possible interference of blue light on the circadian rhythm [91] resulted in reduced inactivity, with a greater display of active behaviours. In the case of *T. gondii+*CL mice, the influence of infection and sickness behaviour led to an increase in inactivity [5,6] resulting in a reduction in active behaviours.

Food and water consumption increased in individuals exposed to BLLT, and this increase has already been observed in another study evaluating the physiological parameters of animals under blue LED light, suggesting a relationship with increased welfare levels [67]. In contrast, changes in eating behaviour, whether hyperphagia (increased food intake) or hypophagia (reduced food intake), have also been reported as responses to chronic stress [103–106], while polydipsia (excessive water consumption) has already been observed in stressed individuals [97]. Although these behaviours may have been expressed more frequently due to increased activity observed in this group, it is important to consider that other behavioural changes were also recorded. Therefore, BLLT should be interpreted with caution, as the behavioural findings suggest a potential stress-related effect under the experimental conditions tested.

Attending behaviour was less observed in BLLT-exposed mice, possibly due to visual discomfort or retinal sensitivity to blue light [98,99]. However, this remains a hypothesis that needs targeted investigation. In the present study, blue light may have interfered with the mice’s visual capacity, as it has the potential to damage their eyes, leading the animals to become more active to avoid the light or more inactive (standing still with their eyes closed, avoiding the light). In both cases, increased or decreased activity reduced the animals’ attention to the environment. However, because this behaviour was rarely observed during the study, further experiments are needed to evaluate this hypothesis.

During the experiment, some infected individuals died in both the exposed to conventional light and the exposed to BLLT groups. The corpses were removed the next day (during the inspection of the boxes), and consequently, other individuals encountered them. While in the infected exposed to the conventional light group (*T. gondii*+CL), there was no relevant interaction (some individuals investigated and slept on top of the corpses), in the infected exposed to the BLLT group (*T. gondii*+BL), cannibalism of the bodies was observed. The interactions with the corpses (mainly investigation and cannibalism) were classified within the “other behaviours” category, which largely explains the higher frequency of this behavioural class in the *T. gondii*+BL group. Various factors, including environmental stressors, can cause cannibalism [110,111], consistent with the possibility that BLLT exposure contributed to stress-related behavioural responses in the animals.

This result may suggest that exposure to light, particularly high-intensity blue light, can act as a chronic stressor, influencing behaviour and welfare in infected individuals. Although speculative, environmental light conditions may also influence host physiological responses in vulnerable populations. However, because natural sunlight differs substantially from controlled BLLT exposure in wavelength composition, irradiance, and duration, no direct extrapolation can be made from the present findings. Blue light, whether artificial or natural, has been shown to influence circadian rhythms, stress hormone levels, and behavioural responses in mammals [100,101]. Prolonged or intense exposure may act as a chronic stressor, potentially exacerbating physiological and behavioural alterations in individuals already affected by parasitic infections, such as *T. gondii*. In humans and animal models, chronic stress has been linked to immune dysregulation, increased neuroinflammation, and poorer disease outcomes [102,103]. Although blue light is present in natural sunlight, exposure to solar radiation, which spans the full wavelength range, is not equivalent to controlled exposure to a specific blue wavelength. Under experimental conditions, parameters such as wavelength, irradiance, exposure duration, and target area can be precisely controlled, producing effects likely distinct from those elicited by ambient sunlight. Therefore, while environmental light may influence host-pathogen dynamics, the effects of selected blue light exposure cannot be directly extrapolated from natural sunlight, underscoring the need for further studies to investigate both artificial and environmental light as modulators of disease progression.

Some mechanisms have been suggested to explain the effects of blue light on parasites, although they remain incompletely characterised. Although the presence and responsiveness of endogenous chromophores in protozoan parasites such as *T. gondii* are not yet fully elucidated, a dual-mechanism model is biologically plausible. First, a direct photodynamic effect may occur, since blue light (e.g., ~ 400–460 nm) can photoexcite intracellular porphyrins or flavins, generating reactive oxygen species (ROS) [104] that damage lipids, proteins, and DNA [105], thereby compromising parasite viability [106–108]. Evidence from other unicellular eukaryotes, such as microsporidia, suggests that protozoan parasites are sensitive to photo-oxidative damage [109]. Moreover, ultrastructural analyses demonstrated that blue‑LED exposure induced pronounced disorganisation and degradation of intracellular organelles in *Leishmania* spp., as revealed by transmission electron microscopy, further supporting a direct photodynamic mode of action [110].

Second, blue light may also act indirectly via host-mediated pathways, particularly by modulating physiological and immunological responses. For example, in our study, blue light exposure modulated oxidative stress markers [75], consistent with the idea that the host’s systemic environment can influence parasite survival. Analogous mechanisms have been observed in other protozoan infections, such as *T. cruzi*, in which light-based treatments reduced parasite burden as quantified by qPCR [47]. Although complementary immune assays, such as IFN-γ or TNF-α gene expression profiling and direct quantification of ROS, would further strengthen mechanistic confirmation, these analyses were beyond the scope of the present behaviourally focused trial and are being addressed in ongoing follow-up work. Additionally, clinical phototherapies, such as those used to treat neonatal jaundice, demonstrate that blue-green light can modify circulating molecules (e.g., bilirubin), thereby facilitating their excretion and preventing tissue damage [111,112]. In parallel, our recently published study demonstrated that blue light exposure reduced cerebral parasite burden and modulated oxidative stress markers (TBARS and SOD), ferritin levels, and tissue-specific cytokine responses during *T. gondii* infection [75]. Together, these findings support the biological plausibility that blue light may exert indirect, host-mediated effects that influence parasite survival in deeper tissues, including the brain. Nevertheless, the precise physiological and immunological pathways involved remain to be fully elucidated.

Therefore, a combined model involving both direct photodynamic damage and indirect host-mediated effects appears to be the interpretation most consistent with the currently available evidence. Nonetheless, the physiological and immunological pathways underlying these effects remain to be fully elucidated and will constitute the next phase of investigation by our research group. Accordingly, upcoming studies will incorporate targeted immunological and oxidative-stress assays (e.g., cytokine profiling and ROS-related pathways) to disentangle direct photodynamic effects from host-mediated mechanisms.

In this study, we also evaluated parasite load and selected oxidative stress parameters. The results from qPCR analyses indicate that blue LED light treatment (BLLT) was associated with reduced parasite load in brain tissue and modulation of specific immunological responses [75]. These findings suggest potential therapeutic value that requires further validation in larger, longer-term experimental studies. However, these parasitological and immunological effects were not mirrored at the behavioural level in our study, suggesting a possible dissociation between parasite reduction and the behavioural alterations associated with *T. gondii* infection [12]. This highlights the need for further investigations to refine phototherapy protocols, aiming to develop approaches that are not only more effective in controlling infection but also less detrimental to the welfare of treated individuals. To our knowledge, this represents the first comprehensive evaluation of the effects of blue light therapy on *T. gondii*, integrating *in vitro* and *in vivo* approaches with behavioural assessments. Mechanisms underlying these effects remain unknown and will be the focus of future investigations by our group. Given that this was a first experimental trial, future protocol adjustments will aim to mitigate stress-related behavioural responses observed here.

Despite the insights gained, this study has several limitations. Firstly, the experimental design focused on the host-parasite interaction without fully accounting for the complex, interconnected system in which this relationship occurs. The concept of the holobiont, which considers the host as a dynamic ecosystem comprising its own cells, microbiota, and genetic background, highlights that infection outcomes and behavioural responses are influenced by multiple interacting factors beyond the parasite itself [113–115]. Secondly, the 11-day observation period was sufficient to evaluate acute behavioural effects in the context of congenital infection, but it may be insufficient to capture chronic-phase behaviours or neuroinflammatory consequences, particularly given the ME49 strain’s Central Nervous System tropism [116]. Thirdly, although behavioural alterations were systematically recorded, additional physiological and molecular markers, including neuroinflammatory and microbiome analyses, were not measured in this study. Fourthly, the effects of *T. gondii* infection itself, as well as potential stress induced by blue LED light therapy, complicate the interpretation of observed behavioural changes. Although irradiation was restricted to the light phase of the photoperiod, we acknowledge that blue light exposure may still modulate circadian-related physiology, and subtle chronobiological effects cannot be entirely excluded without direct measurements. Moreover, because light exposure alone is known to influence immune and stress physiology in healthy animals, part of the response observed in the present study may reflect a general photobiological effect rather than an infection-specific phenomenon, a consideration that should be kept in mind when interpreting the findings in the context of previous phototherapy research. Additionally, because this study was conducted exclusively in pregnant Swiss Webster mice, the behavioural and physiological responses observed here may be species- and condition-specific, and extrapolation to other host models or taxa should be made with caution. Finally, there is a lack of direct clinical or behavioural correlation with parasite reduction, which limits the strength of conclusions regarding therapeutic efficacy. In addition to these factors, the relatively small sample size in each experimental group represents a significant limitation. This issue was further compounded by infection-related mortality (four animals across the infected groups), an inherent challenge in in vivo studies involving parasitic pathogens. This issue may have been further amplified by the use of pregnant animals, whose altered immunophysiological state can increase susceptibility to acute toxoplasmosis and contribute to greater variability in behavioural and clinical outcomes. Because these deaths occurred during the final two days of the experiment, most behavioural data had already been collected and could be retained in the analyses. Moreover, the use of GLMMs allowed repeated behavioural observations from the same individuals to be incorporated while partially accounting for inter-individual variability and unbalanced sampling resulting from mortality events. Nevertheless, the loss of nearly half of the infected animals reduced statistical power for comparisons at the latest time points and may have introduced survivorship bias, as behavioural outcomes at the end of the observation period necessarily reflect only the individuals that survived the acute phase of infection. Thus, behavioural comparisons should be interpreted with caution, and future studies employing larger cohorts and improved survival-support strategies are recommended.

Accordingly, although the findings provide valuable preliminary insights, they must be interpreted with caution, particularly regarding behavioural analyses. Future studies employing larger cohorts, extended monitoring periods, and improved survival-support strategies are recommended, in full accordance with ethical guidelines and the logistical constraints of research involving infectious agents. Collectively, these limitations underscore the need for cautious interpretation of our findings and emphasise the importance of adopting a more comprehensive, multidisciplinary approach for future investigations.

## Conclusion

The blue light (470nm), for 12 12-hour/day regime, may have triggered stress in the confined mice, which was associated with behavioural responses suggestive of stress, such as increased allogrooming, increased display of abnormal behaviours in infected animals, increased food and water intake, and the emergence of other behaviours such as cannibalism. Overall, these responses suggest that prolonged exposure to blue light under the tested conditions may influence welfare-related behavioural parameters in the animals, although these findings should be interpreted with caution given the exploratory nature and sample size limitations of the study. In addition, there was no significant difference between the infected groups in the display of behaviours indicative of sickness behaviour, and deaths occurred in both groups, which suggests that the behavioural effects of the treatment did not translate into clear improvements in sickness-related behavioural outcomes during the observation period. However, the qPCR results showed that the infected group exposed to the treatment (*T. gondii* + BL) exhibited a significant reduction in parasite load, particularly in the brain [75]. Therefore, BLLT showed evidence of antiparasitic effects at the molecular level, although these effects were not accompanied by corresponding improvements in behavioural outcomes under the present experimental conditions. However, these findings are based on a single murine model and should not be directly generalised to other host species without further validation in complementary experimental systems.

Additional physiological and immunological findings from the same experimental trial, including oxidative stress and cytokine-related markers, have been reported elsewhere [75] and provide complementary context for interpreting the behavioural and parasitological findings discussed here. We encourage further research to better evaluate the safety, efficacy, and mechanistic basis of this therapeutic approach. Future studies should incorporate direct chronobiological assessments, such as locomotor activity monitoring, corticosterone profiling, and/or clock-gene expression analyses, to better determine whether blue light exposure induces subtle circadian modulation and to disentangle phototherapeutic effects from potential rhythm-related behavioural changes. However, we emphasise the importance of reviewing the blue LED light application regimen, as behavioural indicators suggest a potential negative effect on welfare-related parameters in treated animals.

Finally, future studies should also adopt a more holistic, multidisciplinary approach to fully understand the effects of blue LED light therapy during *T. gondii* infection. Such research should integrate analyses of neuroinflammatory markers, gut microbiome composition, immune modulation, and the long-term impact of light exposure on behaviour and welfare. Investigating different blue light regimens may help determine whether antiparasitic effects can be maintained while reducing potential stress-related behavioural impacts. Additionally, assessing the impact of chronic exposure to natural light, particularly in tropical regions where toxoplasmosis is endemic, could help clarify whether environmental light serves as a stressor influencing disease progression. By embracing the complexity of the host-parasite-environment interaction, future studies will provide a better understanding of safe and practical applications of phototherapy for parasitic infections.

## Supporting information

S1 File Supporting Information: Supplementary methods describing the real-time quantitative PCR (qPCR) assay used to confirm *Toxoplasma gondii* infection and quantify parasite burden, together with the corresponding supplementary results comparing parasite load between treatment groups and tissues. (PDF)

S2 File Supporting Information: Photograph of the experimental apparatus used to deliver blue LED light therapy while simultaneously recording animal behaviour. (PDF)

S3 File Supporting Information: Descriptive statistics for the behaviours recorded for each treatment (NInf+ + CL: non-infected exposed to conventional light; NInf+ + BL: non-infected exposed to treatment with blue LED light; *T. gondii*+CL: infected with *T. gondii* exposed to conventional light; *T. gondii*+BL: infected with *T. gondii* exposed to treatment with blue LED light). (SE: Standard Error; Std.Dev: Standard Deviation; Min: Minimal Value; 1Q: first quartile; 3Q: third quartile; Max: Maximal Value; GRO: Grooming; INA: Inactive; AFI: Affiliative Interactions; AGI: Agonistic Interactions; ABN: Abnormal Behaviours; ACT: Active; DR: Drinking; FE: Feeding; ATT: Attend; OTH: Others).
(PDF)

S4 File Supporting Information: Heatmap of behavioural activity across experimental treatments. Heatmap showing the mean (± SD) total number of behavioural records per individual for each behavioural category across the four experimental treatments: non-infected exposed to conventional light (NInf + CL), non-infected exposed to blue LED light therapy (NInf + BL), *T. gondii*-infected exposed to conventional light (*T. gondii*+CL), and *T. gondii*-infected exposed to blue LED light therapy (*T. gondii*+BL). Behavioural categories include grooming (GRO), inactivity (INA), affiliative interactions (AFI), agonistic interactions (AGI), abnormal behaviours (ABN), active behaviours (ACT), drinking (DR), feeding (FE), attending behaviour (ATT), and other behaviours (OTH). Values displayed inside each cell correspond to the mean ± standard deviation of behavioural counts pooled across the experimental observation period. Cell colours represent the relative intensity of each behaviour after row-wise z-score standardisation, allowing comparison of behavioural profiles across treatments. The colour scale (approximately −0.5 to 2.5) indicates how much each treatment deviates from the overall behavioural mean for that specific category, with higher positive values representing relatively increased behavioural expression. Hierarchical clustering dendrograms shown along the margins group behaviours and treatments according to similarity in their behavioural patterns, highlighting clusters of responses potentially associated with infection status and/or blue light exposure.
(PDF)

S5 File Supporting Information: Generalised Linear Mixed Models (GLMM) results for all behaviours expressed by mice (*Mus musculus*) depending on the treatment. Data were analysed using GLMMs with negative binomial distribution, treatment as fixed factor and experimental day as random factor. Deviance, degrees of freedom (DF), Chi-square (Chisq) and exact p-values are shown for the comparison between the full model (Treatment + Day) and the null model (Day only). Marginal R² represents the variance explained by fixed effects only (treatment), while conditional R² represents the variance explained by both fixed and random effects (treatment + day). Rate ratios (exp(estimate)) with 95% confidence intervals (CI) are presented for each treatment group compared to the control (NN: Non-infected + Conventional Light). Agonistic Interactions were excluded from the analysis due to an excessive number of zero counts (convergence failure). Drinking and Attend models showed singular fit, meaning that the random effect variance (Day) was close to zero; therefore, R² values could not be reliably calculated. Abbreviations: NN = Non-infected + Conventional Light (NInf + CL); NS = Non-infected + BLLT (NInf + BL); SN = *T. gondii* infected + Conventional Light (*T. gondii* + CL); SS = *T. gondii* infected + BLLT (*T. gondii* + BL). Significance levels: *** p < 0.001; ** p < 0.01; * p < 0.05. Interpretation of Rate Ratio: Rate ratio > 1 indicates the behaviour increased compared to the control group (NN); rate ratio < 1 indicates the behaviour decreased compared to the control group (NN).
(PDF)
