# Supplementary material for: Interference of phototherapy with blue LED light on the behaviour of mice infected with Toxoplasma gondii
Source: PLoS One. 2026 Jul 14;21(7):e0353740. doi: 10.1371/journal.pone.0353740 (PMC13367692; doi:10.1371/journal.pone.0353740)
Supplement: S1 File — (PDF) [file pone.0353740.s001.pdf]

S1 Supporting Information

Supplementary Methods

Real-time Quantitative PCR (qPCR)

To confirm infection and compare parasite burden between treatment groups, a quantitative PCR (qPCR) assay targeting the multicopy *T. gondii* REP-529 element (112 bp; GenBank AF487550) was performed on genomic DNA extracted from maternal brain and placental tissues. Sample processing and DNA extraction followed the procedures described in Toledo et al. (2026).

Each reaction contained 10 µL of GoTaq® qPCR Master Mix (Promega), 1 µL of each primer (forward: 5'-AGAGACACCGGAATGCGATCT-3'; reverse: 5'-TTCGTCCAAGCCTCCGACT-3'), 3 µL of template DNA, and nuclease-free water to a final volume of 20 µL. Amplifications were carried out on an ABI 7500 Real-Time System (Applied Biosystems) using the following cycling conditions: initial denaturation at 95 °C for 2 minutes, followed by 40 cycles of 95 °C for 15 seconds and 60 °C for 1 minute. Parasite load was expressed as tachyzoite equivalents per microgram of total DNA.

Data distribution was evaluated using the Kolmogorov–Smirnov test. Since the data were non-normally distributed, comparisons between light-exposed infected groups (*T. gondii* + CL vs. *T. gondii* + BL) were performed using Mann–Whitney U tests. The same test was used to contrast parasite load between brain and placental samples under each lighting condition.

Results

Real-time qPCR Analysis

All analysed tissues tested positive for *T. gondii*, confirming successful establishment of infection. Parasite quantification revealed a markedly lower burden in the brains of

females exposed to blue LED light therapy (*T. gondii* + BL) compared with infected animals maintained under conventional lighting (*T. gondii* + CL) (U = 1, p = 0.008239).

In placental tissues, however, no difference in parasite load was detected between the two infected groups (U = 8, p = 0.1282). Across both lighting conditions, parasite levels were consistently higher in brain tissue than in placenta (conventional light: U = 36, p = 0.005075; blue light: U = 36, p = 0.005075).

Reference

Toledo DNM, Carlos N, Dantas-Vieira GC, de Castro Burle MM, Duarte-Junior L de A, Cruz Castro ML, et al. Blue light-emitting diode phototherapy reduces cerebral parasitic load and inflammation in murine congenital toxoplasmosis model. Immunobiology. 2026;231: 153182. doi:10.1016/j.imbio.2026.153182
