# Supplementary figures and images for: Interference of phototherapy with blue LED light on the behaviour of mice infected with Toxoplasma gondii
Source: PLoS One. 2026 Jul 14;21(7):e0353740. doi: 10.1371/journal.pone.0353740 (PMC13367692; doi:10.1371/journal.pone.0353740)

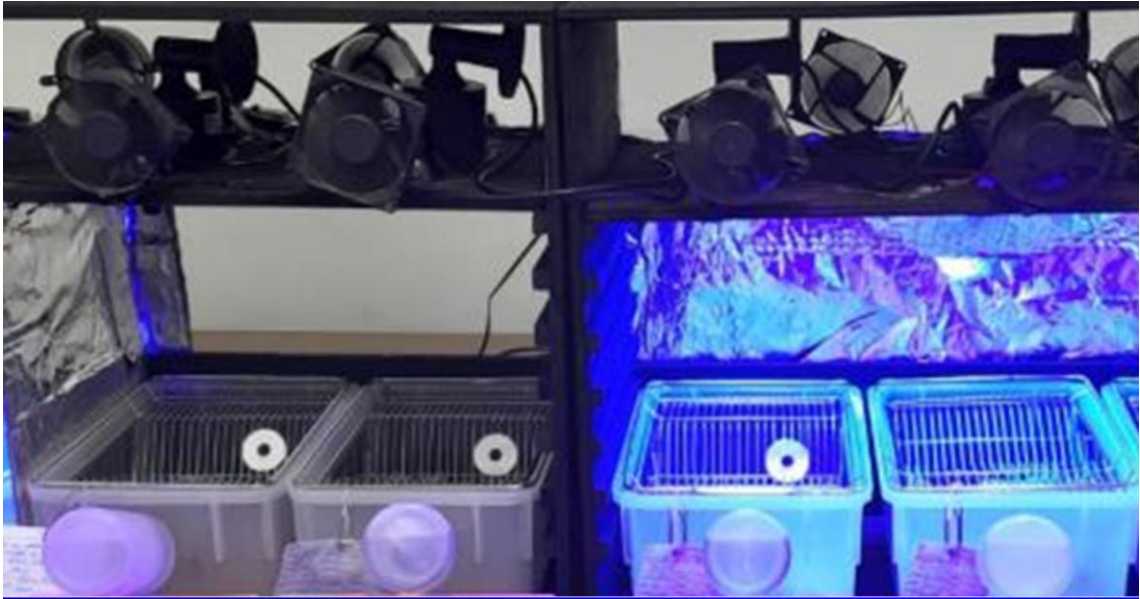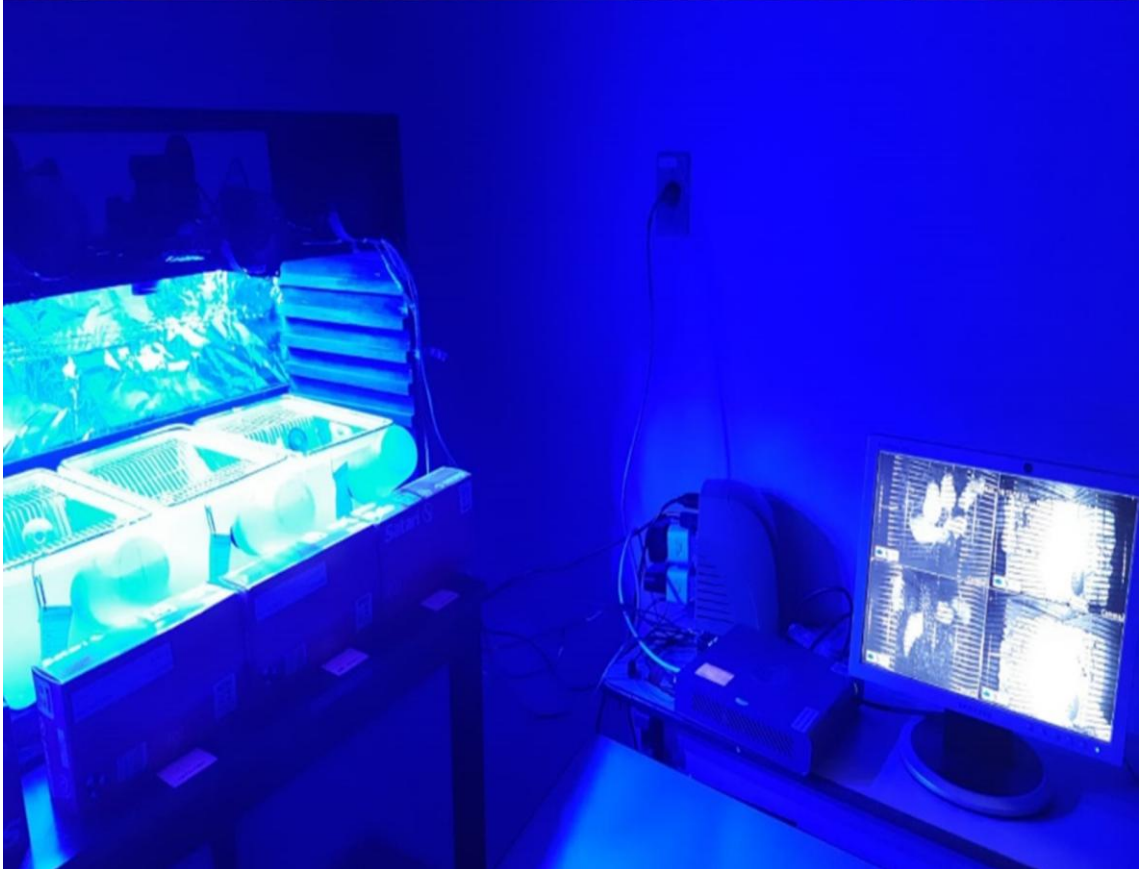

**S2 Supporting Information.**

Supplement: S2 File — (PDF) [file pone.0353740.s002.pdf]

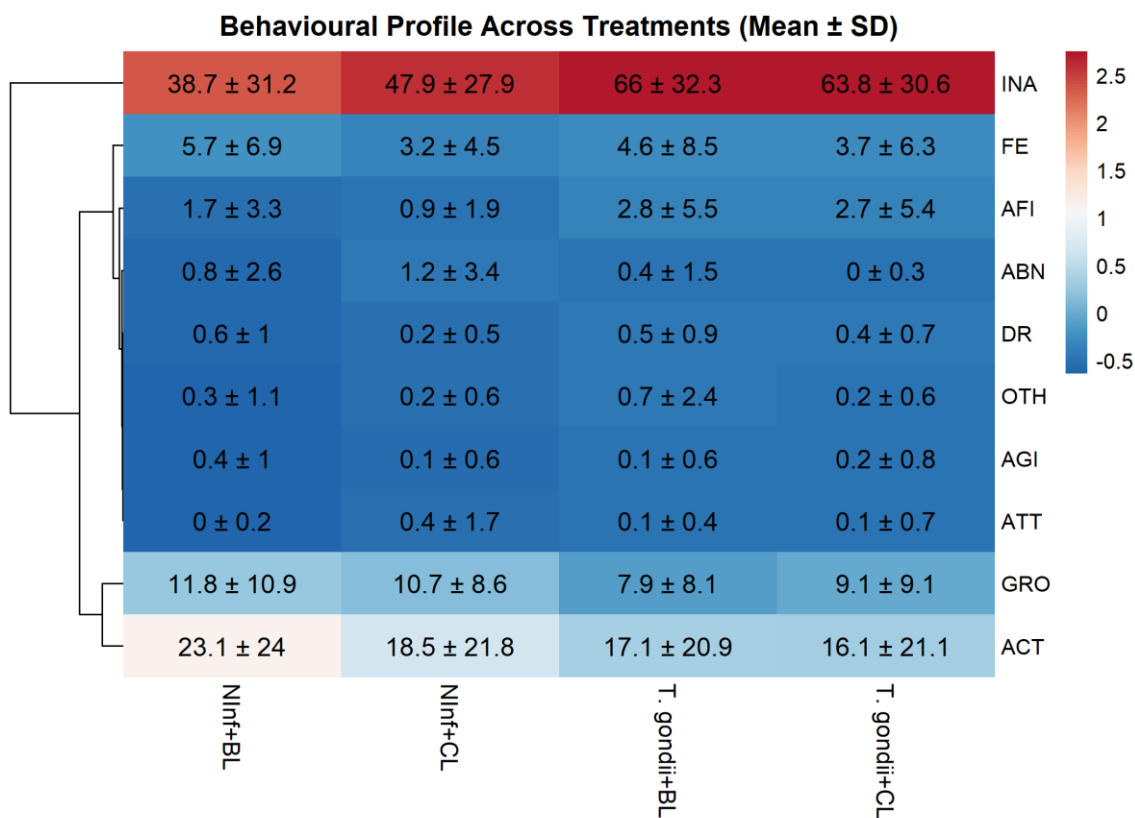

**S4 Supporting Information.**

Supplement: S4 File — Heatmap showing the mean (± SD) total number of behavioural records per individual for each behavioural category across the four experimental treatments: non-infected exposed to conventional light (NInf + CL), non-infected exposed to blue LED light therapy (NInf + BL), T. gondii-infected exposed to conventional light (T. gondii+CL), and T. gondii-infected exposed to blue LED light therapy (T. gondii+BL). Behavioural categories include grooming (GRO), inactivity (INA), affiliative interactions (AFI), agonistic interactions (AGI), abnormal behaviours (ABN), active behaviours (ACT), drinking (DR), feeding (FE), attending behaviour (ATT), and other behaviours (OTH). Values displayed inside each cell correspond to the mean ± standard deviation of behavioural counts pooled across the experimental observation period. Cell colours represent the relative intensity of each behaviour after row-wise z-score standardisation, allowing comparison of behavioural profiles across treatments. The colour scale (approximately −0.5 to 2.5) indicates how much each treatment deviates from the overall behavioural mean for that specific category, with higher positive values representing relatively increased behavioural expression. Hierarchical clustering dendrograms shown along the margins group behaviours and treatments according to similarity in their behavioural patterns, highlighting clusters of responses potentially associated with infection status and/or blue light exposure. (PDF) [file pone.0353740.s004.pdf]
