# Supplementary material for: Interference of phototherapy with blue LED light on the behaviour of mice infected with Toxoplasma gondii
Source: PLoS One. 2026 Jul 14;21(7):e0353740. doi: 10.1371/journal.pone.0353740 (PMC13367692; doi:10.1371/journal.pone.0353740)
Supplement: S3 File — (SE: Standard Error; Std.Dev: Standard Deviation; Min: Minimal Value; 1Q: first quartile; 3Q: third quartile; Max: Maximal Value; GRO: Grooming; INA: Inactive; AFI: Affiliative Interactions; AGI: Agonistic Interactions; ABN: Abnormal Behaviours; ACT: Active; DR: Drinking; FE: Feeding; ATT: Attend; OTH: Others). (PDF) [file pone.0353740.s003.pdf]

S3 Supporting Information.

Variable	Treatment	Mean	SE	Std.Dev.	Variance	Min	1Q	Median	3Q	Max
**GRO**	NInf + CL	10.719	0.692	8.563	73.322	0.000	3.000	9.000	15.000	38.000
	NInf + BL	11.830	0.879	10.878	118.326	0.000	3.000	9.000	16.500	81.000
	T. gondii + CL	9.078	0.735	9.093	82.678	0.000	2.000	6.000	13.000	41.000
	T. gondii + BL	7.876	0.657	8.128	66.070	0.000	2.000	6.000	10.500	49.000
**INA**	NInf + CL	47.86	2.25	27.88	777.57	0.00	27.00	54.00	72.00	84.00
	NInf + BL	38.71	2.52	31.22	974.75	0.00	3.50	44.00	69.00	84.00

	T. gondii + CL	63.85	2.47	30.57	934.48	0.00	43.00	65.00	92.00	105.00
	T. gondii + BL	66.04	2.61	32.29	1042.83	0.00	51.50	77.00	90.00	105.00
**AFI**	NInf + CL	0.902	0.151	1.874	3.510	0.000	0.000	0.000	1.500	10.000
	NInf + BL	1.745	0.264	3.264	10.652	0.000	0.000	0.000	2.000	18.000
	T. gondii + CL	2.673	0.437	5.411	29.274	0.000	0.000	0.000	3.000	34.000
	T. gondii + BL	2.837	0.445	5.509	30.348	0.000	0.000	0.000	4.000	31.000
**AGI**	NInf + CL	0.1176	0.0499	0.6171	0.3808	0.0000	0.0000	0.0000	0.0000	6.0000
	NInf + BL	0.3529	0.0832	1.0290	1.0588	0.0000	0.0000	0.0000	0.0000	8.0000
	T. gondii + CL	0.1699	0.0614	0.7591	0.5762	0.0000	0.0000	0.0000	0.0000	6.0000

	T. gondii + BL	0.1046	0.0446	0.5521	0.3048	0.0000	0.0000	0.0000	0.0000	4.0000
**ABN**	NInf + CL	1.242	0.273	3.382	11.435	0.000	0.000	0.000	0.000	20.000
	NInf + BL	0.850	0.208	2.569	6.602	0.000	0.000	0.000	0.000	17.000
	T. gondii + CL	0.0327	0.0235	0.2906	0.0845	0.0000	0.0000	0.0000	0.0000	3.0000
	T. gondii + BL	0.366	0.118	1.459	2.128	0.000	0.000	0.000	0.000	11.000
**ACT**	NInf + CL	18.53	1.76	21.81	475.51	0.00	2.00	9.00	30.00	77.00
	NInf + BL	23.05	1.94	24.03	577.38	0.00	3.50	13.00	37.50	84.00
	T. gondii + CL	16.08	1.71	21.15	447.28	0.00	3.00	9.00	20.50	103.00
	T. gondii + BL	17.06	1.69	20.92	437.62	0.00	3.00	9.00	21.50	92.00
**DR**	NInf + CL	0.2484	0.0418	0.5166	0.2669	0.0000	0.0000	0.0000	0.0000	2.0000

	NInf + BL	0.6405	0.0768	0.9502	0.9028	0.0000	0.0000	0.0000	1.0000	4.0000
	T. gondii + CL	0.3660	0.0562	0.6954	0.4836	0.0000	0.0000	0.0000	1.0000	3.0000
	T. gondii + BL	0.5490	0.0742	0.9172	0.8413	0.0000	0.0000	0.0000	1.0000	5.0000
**FE**	NInf + CL	3.170	0.362	4.478	20.050	0.000	0.000	1.000	5.500	20.000
	NInf + BL	5.719	0.560	6.931	48.045	0.000	0.000	3.000	10.000	30.000
	T. gondii + CL	3.699	0.511	6.320	39.948	0.000	0.000	0.000	5.000	33.000
	T. gondii + BL	4.562	0.685	8.472	71.774	0.000	0.000	0.000	5.500	41.000
**ATT**	NInf + CL	0.366	0.140	1.727	2.984	0.000	0.000	0.000	0.000	17.000
	NInf + BL	0.0261	0.0129	0.1601	0.0256	0.0000	0.0000	0.0000	0.0000	1.0000
	T. gondii + CL	0.0850	0.0564	0.6972	0.4862	0.0000	0.0000	0.0000	0.0000	8.0000

	T. gondii + BL	0.0523	0.0290	0.3589	0.1288	0.0000	0.0000	0.0000	0.0000	4.0000
**OTH**	NInf + CL	0.2288	0.0512	0.6335	0.4013	0.0000	0.0000	0.0000	0.0000	3.0000
	NInf + BL	0.2876	0.0853	1.0555	1.1141	0.0000	0.0000	0.0000	0.0000	9.0000
	T. gondii + CL	0.1765	0.0483	0.5973	0.3568	0.0000	0.0000	0.0000	0.0000	3.0000
	T. gondii + BL	0.699	0.192	2.376	5.646	0.000	0.000	0.000	0.000	21.000
