# Supplementary material for: Interference of phototherapy with blue LED light on the behaviour of mice infected with Toxoplasma gondii
Source: PLoS One. 2026 Jul 14;21(7):e0353740. doi: 10.1371/journal.pone.0353740 (PMC13367692; doi:10.1371/journal.pone.0353740)
Supplement: S5 File — Data were analysed using GLMMs with negative binomial distribution, treatment as fixed factor and experimental day as random factor. Deviance, degrees of freedom (DF), Chi-square (Chisq) and exact p-values are shown for the comparison between the full model (Treatment + Day) and the null model (Day only). Marginal R² represents the variance explained by fixed effects only (treatment), while conditional R² represents the variance explained by both fixed and random effects (treatment + day). Rate ratios (exp(estimate)) with 95% confidence intervals (CI) are presented for each treatment group compared to the control (NN: Non-infected + Conventional Light). Agonistic Interactions were excluded from the analysis due to an excessive number of zero counts (convergence failure). Drinking and Attend models showed singular fit, meaning that the random effect variance (Day) was close to zero; therefore, R² values could not be reliably calculated. Abbreviations: NN = Non-infected + Conventional Light (NInf + CL); NS = Non-infected + BLLT (NInf + BL); SN = T. gondii infected + Conventional Light (T. gondii + CL); SS = T. gondii infected + BLLT (T. gondii + BL). Significance levels: *** p < 0.001; ** p < 0.01; * p < 0.05. Interpretation of Rate Ratio: Rate ratio > 1 indicates the behaviour increased compared to the control group (NN); rate ratio < 1 indicates the behaviour decreased compared to the control group (NN). (PDF) [file pone.0353740.s005.pdf]

S5 Supporting Information

Response Variable	Deviance	DF	Chisq	P-value	Marginal R^2	Condicional R^2	Effect Size (Rate Ration - 95% CI)
**Grooming**	4056.6	3	20.172	0.000156 ***	0.0452	0.0975	NS vs NN: 1.108 [0.896-1.370]; SN vs NN: 0.805 [0.649-0.997]*; SS vs NN: 0.700 [0.564-0.868]**
**Inactive**	17242	3	1451.2	< 0.001 ***	0.5638	0.7822	NS vs NN: 0.809 [0.781-0.837]*; SN vs NN: 1.334 [1.294-1.375]*; SS vs NN: 1.380 [1.339-1.422]***
**Affiliative Interactions**	2006.4	3	17.89	0.000463 ***	0.0859	0.1453	NS vs NN: 2.001 [1.216-3.292]; SN vs NN: 2.728 [1.664-4.470]*; SS vs NN: 2.795 [1.706-4.579]***
**Abnormal Behaviour**	794.9	3	43.692	< 0.001 ***	0.4101	0.5603	NS vs NN: 0.467 [0.190-1.144]; SN vs NN: 0.015 [0.004-0.058]*; SS vs NN: 0.151 [0.056-0.409]*
**Active**	4765.4	3	10.171	0.01716 *	0.0261	0.0689	NS vs NN: 1.249 [0.948-1.646]; SN vs NN: 0.818 [0.620-1.078]; SS vs NN:

Response Variable	Deviance	DF	Chisq	P-value	Marginal R^2	Condicional R^2	Effect Size (Rate Ration - 95% CI)
							0.880 [0.667-1.160]; NS vs SN: 1.528 [1.159-2.014]*
**Drinking**	1077.1	3	23.482	< 0.001 ***	N/A[1]	-	NS vs NN: 2.579 [1.681-3.957]*; SN vs NN: 1.474 [0.929-2.337]; SS vs NN: 2.211 [1.430-3.417]*
**Feeding**	2773.2	3	8.597	0.03517 *	0.0263	0.1783	NS vs NN: 1.651 [1.082-2.517]*; SN vs NN: 0.942 [0.615-1.443]; SS vs NN: 1.089 [0.712-1.665]
**Attend**	298.7	3	12.891	0.00488 **	N/A[1]	-	NS vs NN: 0.071 [0.014-0.353]; SN vs NN: 0.232 [0.059-0.916]*; SS vs NN: 0.143 [0.034-0.602]
**Other**	730.41	3	10.827	0.01270 *	0.0781	0.1345	NS vs NN: 1.222 [0.527-2.837]; SN vs NN: 0.742 [0.308-1.786]; SS vs NN: 2.754 [1.226-6.187]*
